# An Insight Into Lyme Prosthetic Joint Infection in Knee Arthroplasty: A Literature Review

**DOI:** 10.5435/JAAOSGlobal-D-21-00191

**Published:** 2022-02-02

**Authors:** Muzaffar Ali, Anthony O. Kamson, David S. Phillips, Scott G. King

**Affiliations:** From the University of Pittsburgh Medical Center—Pinnacle (Dr. Ali, Dr. Kamson, Dr. Phillips, Dr. King), Harrisburg, PA, and the UPMC Pinnacle—Arlington Orthopedics (Dr. King), Harrisburg, PA.

## Abstract

Lyme prosthetic joint infection (PJI) is a rare event, but it is imperative to include Lyme disease as a possible cause of PJI in a Lyme-endemic region. The purpose of this article was to review the reported cases of Lyme PJIs in knee arthroplasty and to initiate the development of a treatment strategy. We found five cases of Lyme PJI in the literature. All patients lived in the northeastern region of the United States. Four patients were successfully treated with surgical intervention and postoperative antibiotics. One patient was successfully treated with intravenous and oral antibiotics for 6 weeks, without surgical intervention. Synovial fluid Lyme polymerase chain reaction and serological tests were positive in all patients. On follow-up visits, after completion of their treatment, all patients were asymptomatic with a painless functional knee. We recommend considering Lyme disease as a cause of culture-negative PJIs in endemic regions. Additional research is needed to clearly define a treatment algorithm. Based on our literature review, we cannot recommend a single best treatment modality for the treatment of Lyme PJI. However, early irrigation and débridement with administration of postoperative antibiotics may improve early clinical outcomes.

Lyme arthritis is a common late manifestation of Lyme disease. It typically presents with joint pain associated with swelling and erythema. Clinically, it is very difficult to distinguish Lyme arthritis from a native septic joint. As far as we know, there have only been 5 cases of Lyme arthritis associated with total knee prosthetic joint infection (PJI) reported in the literature.^1,2,3,4^ The management of PJI is based on the acuity of symptoms and can include irrigation and débridement, intravenous antibiotics, and implant retention if symptoms are of less than 3 weeks' duration. Chronic infection often requires a two-stage exchange arthroplasty and an extended course of antibiotics.^5^ Diagnosis remains difficult and, when associated with Lyme disease, becomes very complicated. The Musculoskeletal Infection Society criteria are often used to assist in the diagnosis of PJI.^6^ Timely diagnosis and early intervention decrease the morbidity and mortality associated with PJI.^5-7^ If treatment is delayed, Lyme disease, caused by the spirochete *Borrelia burgderfori*, can lead to the formation of biofilms and degradative matrix metalloproteinase enzymes that can lead to implant failure and loosening.^8-10^ However, most reported Lyme PJI cases have been treated with an early irrigation and débridement with postoperative antibiotics. An early treatment is crucial to decrease the bacterial burden to minimize the adverse outcomes of PJI.^1,2,3,4,5,7^

## Methods

The literature review was done using Scopus and PubMed published reports of cases of PJIs secondary to Lyme disease. Search terms included “Lyme,” “Lyme disease,” “culture negative,” “total knee arthroplasty,” “TKA,” and “prosthetic joint infection.” Only five reported cases were encountered. Each reported case was analyzed for diagnostic protocol, knee aspiration results, type of surgical intervention, antibiotics administered, and patient outcome.

### Case 1

A 67-year-old male avid huntsman from a Lyme-endemic region underwent a left medial compartment unicompartmental joint arthroplasty 1 year before presentation.^1^ He reported of 3 months of pain and swelling. Clinical examination revealed a moderate joint effusion, but no erythema or notable pain with range of motion. Serum laboratory results showed erythrocyte sedimentation rate (ESR) 25 mm/hr, C-reactive protein (CRP) 0.7 mg/dL, and positive *B. burgdorferi* antibody enzyme-linked immunofluorescence assay (ELISA) test. A knee aspiration revealed purulent fluid with 51,543 cells/µL, 91.8% neutrophils, positive human neutrophil elastase and α-defensin, elevated CRP, and positive Lyme polymerase chain reaction (PCR). Synovial fluid Gram-stain and bacterial cultures were negative. The patient was started on oral doxycycline and was then converted to intravenous ceftriaxone for 6 weeks. Surgical intervention was not done, given the patient's notable clinical improvement after the initiation of antibiotics. Synovial fluid analysis after the completion of antibiotic therapy demonstrated 540 cells/µL, 38% neutrophils, negative human neutrophil elastase, negative α-defensin, normal CRP, and negative Lyme PCR. Repeat aspiration cultures were negative, and serum ESR and CRP were within normal limits.

### Case 2

An 89-year-old woman with a history of a right total knee arthroplasty (TKA) done 16 years earlier presented to the emergency department with complaints of right knee pain, swelling, and stiffness for the last day.^2^ She did report a dental cleaning 1 month earlier, without antibiotic prophylaxis. On examination, she had a right knee effusion with limited range of motion due to a pain. All vital signs were within normal limits. Laboratory data included a serum white blood count of 13.4 × 1,000/μL, 74% neutrophils, CRP of 10.1 mg/dL, and ESR of 19 mm/hr. A urinalysis confirmed bacteriuria. A right knee aspiration demonstrated 66,100 cells/μL, with 93% neutrophils, negative Gram-stain, and presence of calcium pyrophosphate crystals.

The patient was started on vancomycin, ceftriaxone, and colchicine for the treatment of presumed PJI and concomitant pseudogout. The next day, she underwent a right knee irrigation and débridement with polyethylene exchange. Intraoperatively, no purulence was noted, and all components were well fixed. Synovial fluid and tissue cultures were negative. Serum Lyme tests showed a positive immunoglobulin M (IgM) and negative IgG. Admission arthrocentesis fluid was reprocessed, and Lyme PCR was found to be positive. Intravenous antibiotics were discontinued, and the patient completed a 28-day course of oral doxycycline. At her 6-week follow-up, the patient's knee pain and swelling had completely resolved. She died 5 months later from heart disease.

### Case 3

An 80-year-old woman with a history of a right total knee arthroplasty done 4 months earlier presented to the clinic with proximal tibial erythema and tenderness.^2^ She denied fevers, chills, sweats, or constitutional symptoms. She was prescribed oral cephalexin, which only improved the erythema. A subsequent arthrocentesis revealed 87,830 cells/μL and 93% polys with a negative Gram-stain.

She was admitted to the hospital for a presumed acute PJI. Vitals signs were within normal limits. Intravenous cefazolin was started. Laboratory data included a serum white blood count of 8.8 × 1,000/μL, ESR of 108 mm/hr, and CRP of 16.1 mg/dL. She then underwent right knee irrigation and débridement with polyethylene exchange. Intraoperatively, there was a “small amount of intra-articular purulence and fibrinous exudate with minimal synovial hypertrophy.”^1^ The components were well fixed. Synovial fluid Lyme PCR and serum Lyme ELISA were positive. All preoperative and intraoperative cultures remained negative for bacterial growth. Her antibiotic regimen consisted of intravenous ceftriaxone for the following 4 weeks and then oral doxycycline for an additional 4 weeks. At the 24-month follow-up, the patient remained asymptomatic without pain or functional impairment.

### Case 4

An 83-year-old man underwent a left TKA approximately 6 years before presentation. His symptoms included pain, erythema, and fever for 3 days.^3^ On examination, his knee exhibited a moderate effusion and limited range of motion. Serum inflammatory markers were elevated, with ESR at 64 mm/hr and CRP at 6.7 mg/dL. A knee aspiration revealed a synovial fluid white blood count (WBC) count of 17,370 cells/µL and 92% neutrophils. The synovial fluid culture was negative for bacterial growth. He was treated to have a presumed culture-negative PJI. Given the presence of intraoperative purulence and synovitis, the patient underwent resection arthroplasty with insertion of a static antibiotic spacer. Intraoperative cultures were also negative for bacterial growth.

Further testing revealed a positive Lyme Immunoglobulin G (IgG), negative Lyme IgM, and positive synovial fluid Lyme PCR. Therefore, he was diagnosed with disseminated Lyme disease. He received 6 total weeks of antibiotics. Repeat aspiration done at 8 weeks postoperatively revealed a negative Lyme synovial fluid PCR. He subsequently underwent revision TKA. At the 1-year follow-up, the patient had a functional and painless TKA.

### Case 5

An 81-year-old man with a history of a right total knee arthroplasty done 15 years earlier presented to the emergency department with a 2-day history of right knee pain, swelling, and stiffness.^4^ Clinical examination revealed a moderate joint effusion, tenderness, erythema, and limited knee range of motion. Radiographs demonstrated a stable prosthesis without signs of loosening. Vital signs were within normal limits. Serum laboratory test results demonstrated an ESR of 22 mm/hr and CRP 13.8 mg/dL. A knee aspiration revealed turbid yellow synovial fluid with 79,344 cells/μL, 77% neutrophils, no crystals, and negative cultures. He subsequently underwent arthroscopic irrigation and débridement, synovectomy, and implant retention. Intraoperatively, there was a small amount of purulence. He was started on intravenous vancomycin and cefepime. Intraoperative synovial fluid and tissue cultures remained negative; however, synovial fluid Lyme PCR and serum ELISA were positive.

The patient was placed on oral doxycycline for 4 weeks. Six weeks after discharge, his knee pain and swelling had completely resolved. Repeat knee aspiration demonstrated negative cultures and Lyme titer. At the 4-month follow-up, his knee remained painless. Unfortunately, he developed a disseminated Lyme infection and died 2 months later because of complications of a ruptured brain aneurysm and a stroke secondary to Lyme neuroborreliosis vasculitis.

## Discussion

**Figure 1:**
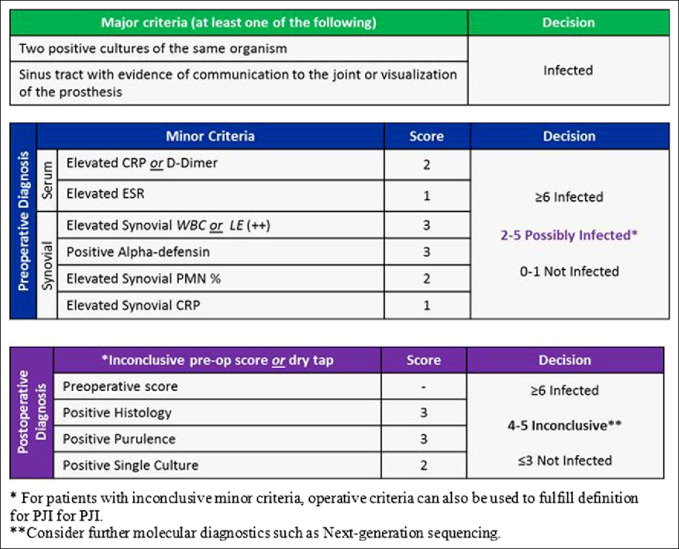
Diagram showing 2018 Musculoskeletal Infection Society criteria for periprosthetic joint infection.

Lyme disease is a result of a bite from the Ixodes tick, allowing the transfer of the spirochete *Borrelia burgdorferi* to another host. The regions most affected by Lyme disease are the Midwestern and Northeastern United States, which includes states such as Pennsylvania, Maine, Illinois, and Minnesota.^11^ In these regions, when a patient presents with an atraumatic knee effusion, Lyme disease should be considered a potential cause. The clinical appearance of Lyme disease is nearly identical to other causes of a septic knee; therefore, it is important to obtain a thorough history to delineate the most likely source of infection.^12^ Late onset arthritis secondary to Lyme disease develops because of hematogenous spread into the synovium, which eventually results in synovitis.^13^ If Lyme disease is refractory to conservative treatment and persistent synovitis is noted, surgical intervention may be indicated to clear the infection.^14-16^ Uncommonly, Lyme disease can cause PJIs. These infections can be very difficult to overcome and place a notable demand on affected patients. PJIs are complex problems requiring prompt diagnosis and treatment to allow for positive outcomes.^7^ The treatment of a Lyme PJI, although rare and likely underreported, remains controversial. Current modalities to treat PJI include a single-stage or a two-stage revision surgery, based on the duration of symptoms and time to evaluation.^6,7^ The Musculoskeletal Infection Society developed criteria, such as positive cultures, presence of a sinus tract, elevated serum CRP and ESR, and synovial fluid laboratory test results, to diagnose PJI and to help surgeons determine if surgical intervention is necessitated as shown in Figure 1.^6^

Another challenge in the setting of a Lyme PJI is the difficulty in culturing *B. burgdoferi*. Because this spirochete is difficult to isolate by cultures alone, a two-step process that includes ELISA and a western blot is commonly used to diagnose Lyme.^17^ To diagnose a Lyme PJI, knee synovial fluid cultures must remain negative for any other organism, but synovial Lyme PCR will result positive. One can only speculate that this could be a common cause of culture-negative PJIs in endemic regions because *B. burgdofderi* would not be noted on routine cultures.^4^ The question arises—should testing for Lyme be routine in the workup of a knee arthroplasty PJI in these regions?^8-10^

We found five cases of Lyme PJI in the current literature (Table 1). All reported cases in the review were subsequently found to be infected with *Borrelia burgdoferi*. All these cases were from the northeastern region of the United States, where there is a high prevalence of Lyme disease. One patient had a unicompartmental knee arthroplasty, whereas the other four patients had total knee arthroplasties. All patients had Lyme disease diagnosed by synovial Lyme PCR and serum ELISA test.^1,2,3,4^ One patient was successfully treated with antibiotics alone.^1^ In the patients treated with surgical intervention, one underwent an arthroscopic irrigation and débridement with synovectomy and implant retention, two patients underwent irrigation and débridement with polyethylene exchange and implant retention, and one underwent a two-stage procedure with resection arthroplasty and antibiotic spacer later followed by a revision total knee arthroplasty. All patients treated with surgical intervention had asymptomatic, functional total knees at their latest documented follow-up.^2-4^

**Table 1:** Reported Lyme Prosthetic Joint Infections and Treatment

Study	Demographics	Procedure	Time Since Procedure	Aspiration	Inflammatory Markers	Surgical Intervention	Antibiotics
Wright and Oliverio^1^	67 M	L mUKA	12 mo	51,543 cells/µL 91.8% nps	ESR 25 mm/hr CRP 0.7 mg/dL	None	Ceftriaxone and doxycycline
Adrados et al^2^	89 F	R TKA	16 yr	66,100 cells/µL 93% nps	ESR 19 mm/hr CRP 10.1 mg/dL	Irrigation and débridement, polyethylene exchange, and antibiotics with implant retention	Doxycycline
	80 F	R TKA	4 mo	87,830 cells/µL 93% nps	ESR 108 mm/hr CRP 16.1 mg/dL	Irrigation and débridement, polyethylene exchange, and antibiotics with implant retention	Ceftriaxone and doxycycline
Collins et al^3^	83 M	L TKA	6 yr	17,370 cells/µL 92% nps	ESR 64 mm/hr CRP 6.7 mg/dL	Resection arthroplasty with antibiotic spacer	Ceftriaxone and doxycycline
Ali et al^4^	81 M	R TKA	15 yr	79,344 cells/µL 77% nps	ESR 22 mm/hr CRP 13.8 mg/dL	Arthroscopic irrigation and débridement, synovectomy, and antibiotics with implant retention	Doxycycline

CRP = C-reactive protein, ESR = erythrocyte sedimentation rate, F = female, L = left, M = male, mUKA = medial unicompartmental knee arthroplasty, nps = neutrophils, R = right, TKA = total knee arthroplasty

Based on the limited available literature, the ideal treatment of postoperative Lyme infection, specifically a Lyme PJI, is still unclear.^18,19^ Wright and Oliverio reported successful treatment with the utilization of only antibiotics.^1^ Adrados et al, Collins et al, and Ali et al reported a combined total of 4 cases treated successfully with surgical intervention. Postoperative antibiotic and doxycycline use was a common factor for the treatment of Lyme PJI in all patients.^2-4^ Based on these findings, we speculate that early irrigation and débridement, antibiotic, and implant retention may be the best option for optimum outcome and fewer long-term complications.^20^ Wright and Oliverio suggested that the ideal treatment of a Lyme PJI may be treatment with antibiotics only; however, they did not have an extended follow-up period to assess the potential long-term complications.^1^ Adrados et al suggested the ideal treatment of Lyme PJI is irrigation and débridement with polyethylene exchange. However, one patient died at 5 months because of coronary artery disease. The second patient did have a follow-up at 24 months where she was asymptomatic with a functional TKA.^2^ The patient reported by Collins et al was treated with a resection arthroplasty and antibiotic spacer. Subsequently, a Lyme PJI diagnosis was made based on negative intraoperative bacterial cultures with a positive Lyme serology from synovial fluid.^3^ Ali et al demonstrated a case where the patient underwent surgical treatment from a Lyme PJI. The patient was completely asymptomatic on his last follow-up and had repeat aspiration cultures and Lyme titer that were negative.^4^ All four patients treated with a form of early surgical intervention demonstrated improved early clinical outcomes with complete resolution of symptoms. Our review has some limitations because two of five patients died within 6 months of their surgery. Only two of five patients have long-term follow-up of 2 years. Both of them were treated surgically and were asymptomatic at their latest follow-up. One patient who was treated with only antibiotics had limited follow-up; therefore, we are unable to assess long-term outcomes with this treatment.

Compared with the nonsurgical treatment, early surgical intervention may be more beneficial in a Lyme PJI because of the ability of *B. burgdorferi* to produce matrix metalloproteinases, which can lead to implant loosening and failure, as well as the fact that Borrelia DNA has been demonstrated to remain in a host even after the completion of an antibiotic therapy.^8,20,21^ Delaying the treatment could lead to persistent bacteria in the joint and eventually the formation of biofilms, making it more difficult to eradicate the infection with antibiotics alone.^8,22^ With early surgical intervention, the amount of time that inflammatory cells accumulate is minimized, which would theoretically lead to decreased metalloproteinase production, in turn causing a decreased likelihood of implant loosening that would cause failure.^21^

The number of total knee arthroplasties done is expected to increase worldwide, and as this number rises, the number of Lyme-related PJIs will likely rise too.^23^ Promptly identifying and treating a complication, such as a PJI, is very important to a patient's quality of life. We believe that Lyme disease should be of higher suspicion as a possible cause of PJI in endemic areas. Acknowledging the available literature, we cannot conclude a single best treatment modality for the treatment of Lyme PJI. However, early irrigation and débridement with antibiotics postoperatively may improve early clinical outcomes.
